# Protocol for the purification of protected carbohydrates: toward coupling automated synthesis to alternate-pump recycling high-performance liquid chromatography†
†Electronic supplementary information (ESI) available: Additional figures and characterization of final compound. See DOI: 10.1039/c6cc07584c



**DOI:** 10.1039/c6cc07584c

**Published:** 2016-10-24

**Authors:** Gabe Nagy, Tianyuan Peng, Daniel E. K. Kabotso, Milos V. Novotny, Nicola L. B. Pohl

**Affiliations:** a Department of Chemistry , Indiana University , Bloomington , IN 47405 , USA . Email: npohl@indiana.edu

## Abstract

Given recent advances in automated oligosaccharide synthesis, analytical techniques that can be coupled to a synthetic framework are needed to not just identify but also purify to homogeneity protected carbohydrate compounds at levels of ≥99.5% purity. Herein, an alternate-pump recycling high-performance liquid chromatography (R-HPLC) method has been developed to allow purification of protected carbohydrates at levels of ≥99.5% purity.

## Abstract 2

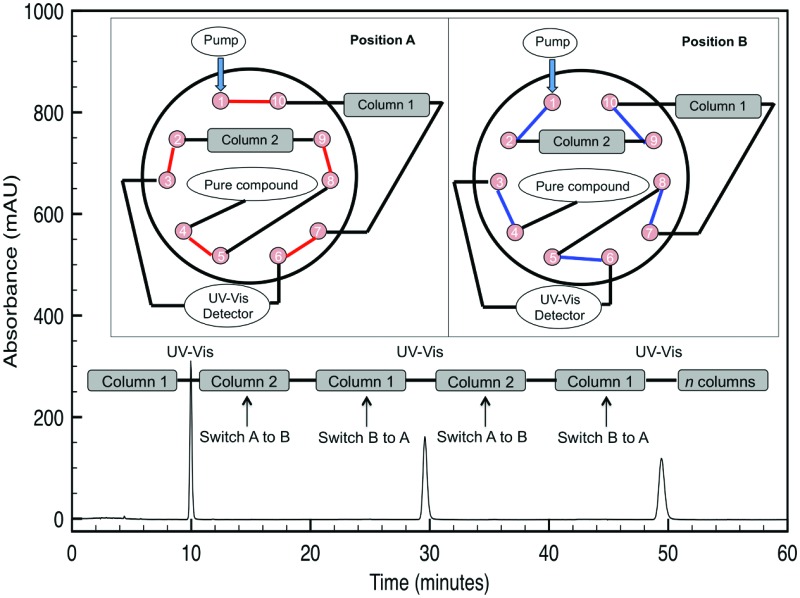
Analytical techniques that can be coupled to automated oligosaccharide synthesis platforms are needed to purify to homogeneity protected carbohydrates at levels of ≥99.5% purity.

The acceptance of automated-peptide synthesis as a valid source of peptides ultimately required the development of C18 reversed-phase chromatography methods.^1^ Unfortunately, such alkyl-linked chromatographic packings are often not sufficient to purify protected carbohydrates (Fig. 1)—which can exist not only as truncated sequences but also as anomeric mixtures and even regioisomers after protecting group migration. Methods to identify impurities^2^ have outstripped abilities to remove those impurities and thereby produce synthetic oligosaccharides at the >99.5% purity needed to avoid potentially lethal adverse effects recently associated with minor impurity contaminants (≤0.5%) present in the leading pharmaceutical heparin sulfate.^3^ Herein is presented the first strategy to achieve such high purity samples of protected oligosaccharides with a method^4^ that can be readily adopted by current HPLC-users. Instead of a direct-pump design where the analyte is required to pass back through the mobile-phase solvent pump, this method uses an alternate-pump design^4^ in which the analyte is recycled between two columns with a 10-port switching valve without passing back through the mobile-phase solvent pump. This newer alternate-pump design^4^ has the advantage over the more common direct-pump design of avoiding unnecessary peak broadening that occurs with the direct-pump design when the analyte is pumped back through the internal volume of the mobile-phase solvent pump. Peak shape is also altered with the direct-pump design because of inversion of the inlet stream concentration profile.^4^ For closely related protected carbohydrate structures, such as anomers or regioisomers, this increase in peak broadening with a direct-pump design may never allow for the separation of the desired product from undesired impurities. Lastly, with carbohydrates being rather “sticky” in their nature, a direct-pump design would directly subject the mobile-phase solvent pump heads to unnecessary wear and tear and potential carryover from prior experiments. For these reasons, we chose to investigate the utility of an alternate-pump recycling HPLC method (Fig. 1)^4^ for separating anomers of protected carbohydrates as a new method to not only identify but also separate synthetic glycans to greater than >99.5% purity—the standard set by pharmaceutical production of heparin sulfate.^3^

**Fig. 1:**
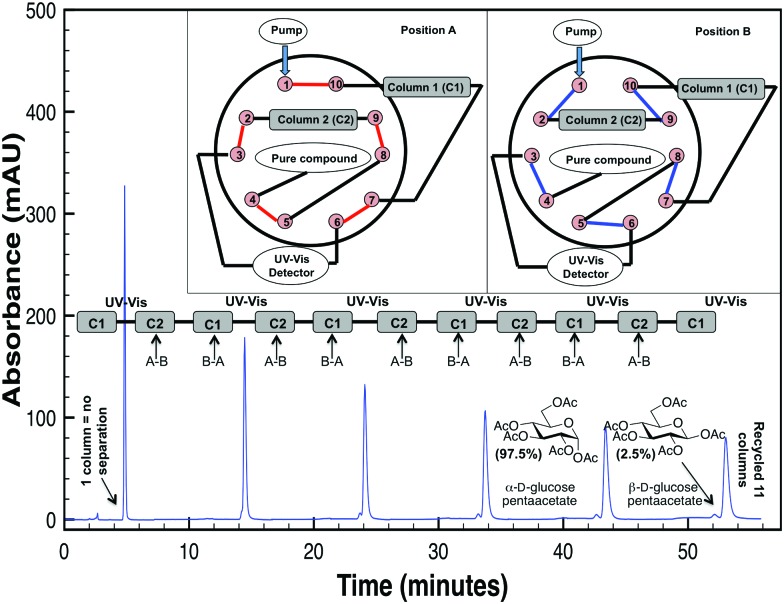
Schematic of alternate-pump R-HPLC with a C5 column chemistry as applied to separate *α*/*β* anomers after 11 total columns. Other peaks are examples of the analyte hitting the UV detector at every odd-numbered effective column.

Alternate-pump recycling high-performance liquid chromatography (R-HPLC), a variant of high-performance liquid chromatography, increases the number of theoretical plates by “recycling” the compound between two identical columns connected by a 10-port switching valve with two positions (A and B) and a UV-detector (Fig. 1). Briefly, the system begins in position A; after a compound is injected, it will flow through the first column until it hits the UV-detector at its retention time (*t*_r_). After the compound is halfway through the second column (1.5*t*_r_), the switching valve will change from position A to B, thereby connecting column 2 directly to column 1. Once the compound travels another column length and is halfway through the first column (2.5*t*_r_), the valve will switch from position B to A, thereby putting it in line with the UV-detector and column 2 again. This process is repeated for as many effective columns as are necessary to provide the desired separation and permit fraction collection since the UV-detector employed is non-destructive in nature. With this set up, the analyte hits the UV detector after every odd-numbered column (1, 3, 5, *etc.*) to generate a spectral peak.

Multiple techniques were considered in the push to achieve ≥99.5% purity of a synthetic protected carbohydrate. Analytical techniques such as nuclear magnetic resonance (NMR), high-resolution mass spectrometry (HRMS), and ion mobility-mass spectrometry (IM-MS) are very useful in providing structural information, but lack the ability to produce a pure sample. Even as strictly a quality control technique for the identification of low-level impurities, NMR cannot detect minor impurities (<∼5%)^5^ and HRMS figures “provide no clues about the purity and which even the clumsiest chemist can readily obtain.^6^” Recently, IM-MS was shown useful in quality control of synthesized carbohydrates at levels ≤1%.^7^ Although in that work compositional isomers, such as trisaccharides with a difference in one monosaccharide unit (d-glucose instead of d-galactose) could not be distinguished, addition of chiral ligands and additives have been shown to discriminate monosaccharides so this approach could potentially be applied to discriminate larger oligosaccharides.^8^ Even so, a major problem with IM-MS as it relates to its ability to act as a quality control technique is the fact that multiple drift time peaks/profiles in a spectrum may not necessarily indicate the presence of two distinct compounds, but rather may indicate the presence of two distinct ion conformations for a single compound.^9^ We still envision current and future IMS-based methods to be very complementary in structural assignment. To drive the acceptance of synthetic oligosaccharides as standards, however, an analytical technique with a non-destructive detector must be selected to produce protected carbohydrates at levels ≥99.5% purity. Single-column reversed-phase high-performance liquid chromatography (HPLC), specifically with alkyl-linked supports, is sometimes used to identify a single peak on a chromatogram,^10^ but the question remains if a single-column injection of a compound can reliably detect impurities at levels ≤0.5%, or if more effective methods are required.

Given the limitations of these common techniques for carbohydrate analysis and the promise of chromatography methods to achieve separations and not just identifications of impurities, we chose to probe the potential of 2D chromatography for the development of a standard for synthetic oligosaccharide purification. Multidimensional chromatography has tremendous potential for complex analyte mixtures and for compounds that cannot be easily separated with one separation mode or stationary phase.^11^ However, the use of 2 or more different column chemistries is problematic because accurate mobile phase adjustments depend on the separation mode used.^12^ Ideally, single column chemistry (stationary phase) could be used, but the column could be of essentially limitless length to guarantee separation of any desired compound. Alternate-pump recycling high-performance liquid chromatography (R-HPLC), a variant of high-performance liquid chromatography, increases the number of theoretical plates by “recycling” the compound between two identical columns and a UV-detector. However, alternate-pump R-HPLC has seen little use to date with only limited applications to isomeric N-glycans and phenylalanine isomers.^4^ In all previous cases, this technique has been employed to separate a very small subsection, or even a single pair, of isomers. With such limited use, it is unclear if a general protocol could be developed and applied for an entire analyte class, such as protected carbohydrates, let alone for high-level purification purposes. The goal here was to develop an alternate-pump R-HPLC-based protocol for the purification of protected carbohydrates at levels ≥99.5%, which mirrors that of heparin sulfate “batch generation on a multi-kilogram scale with a purity higher than 99.5%”.^3^

To this end, several key questions needed to be addressed. First, does our R-HPLC approach increase the separation compared to a single column approach for the commonly used C5 stationary phase; *i.e.* is alternate-pump R-HPLC even really necessary? Since protected carbohydrates are hydrophobic in nature and most protecting groups contain π-bonds, a reversed-phase approach makes sense. However, can other stationary phases such as pentafluorophenyl (PFP) with π–π stacking and dipole–dipole interactions or phenyl hexyl with wedge and skew π–π interactions provide increased separation as compared to the commonly used C5 stationary phase given the ubiquitous benzyl protecting groups in oligosaccharide synthesis? Previous literature has reported that π-bond-containing analytes exhibit increased separation on π-bond-containing reversed-phase stationary phases (such as phenyl hexyl and PFP) when methanol is used instead of acetonitrile as the organic modifier.^13^ A rationale for this outcome is that acetonitrile with its π electron-containing carbon–nitrogen triple bond could disrupt, or even inhibit, the necessary π–π interactions for separation on phenyl hexyl or PFP columns, whereas methanol provides no such deleterious effects.^14^ This asks the question as to whether this result also holds true for protected carbohydrates.

To address the first question, experiments with an isocratic mode (the most straightforward separation mode) single-C5 column HPLC system (the leftmost peak in Fig. 1) showed this system failed to separate minor impurities between peracetylated glucose anomers. However, our R-HPLC system was able to tease apart the two anomers of this protected glucose after 11 total/effective columns as seen by the two peaks at the righthand side of the spectra (Fig. 1; for additional examples, please see the ESI†). With this information in mind, the second question was to determine if a PFP or phenyl hexyl stationary phase could provide increased separation over a C5 one with an our R-HPLC approach. This kind of direct comparison is necessary for a variety of reasons, such as how the functionality of the molecule (*e.g.* what protecting groups are employed), size (*e.g.* monosaccharide *versus* disaccharides, *etc.*), and differences between anomers and regioisomers affect separation in our R-HPLC system. Furthermore, this process was chosen since it was decidedly easier to screen stationary phases for these protected carbohydrate analytes, rather than spend time optimizing conditions for a certain column chemistry that may ultimately remain suboptimal.^14^

Fig. 2 demonstrates cross-column chemistry (stationary phase) comparison between C5, PFP, and phenyl hexyl columns, with various protected carbohydrates that differ both in their number of monosaccharide units, anomericity, and protecting groups. As matching previous experiments,^14^ it was seen that methanol was a superior organic modifier as compared to acetonitrile for both the phenyl hexyl and PFP stationary phases (ESI†). It is important to mention that methanol was not used with the C5 stationary phase based on surpassing of manufacturer recommended pressure limits (∼300 bar).

**Fig. 2:**
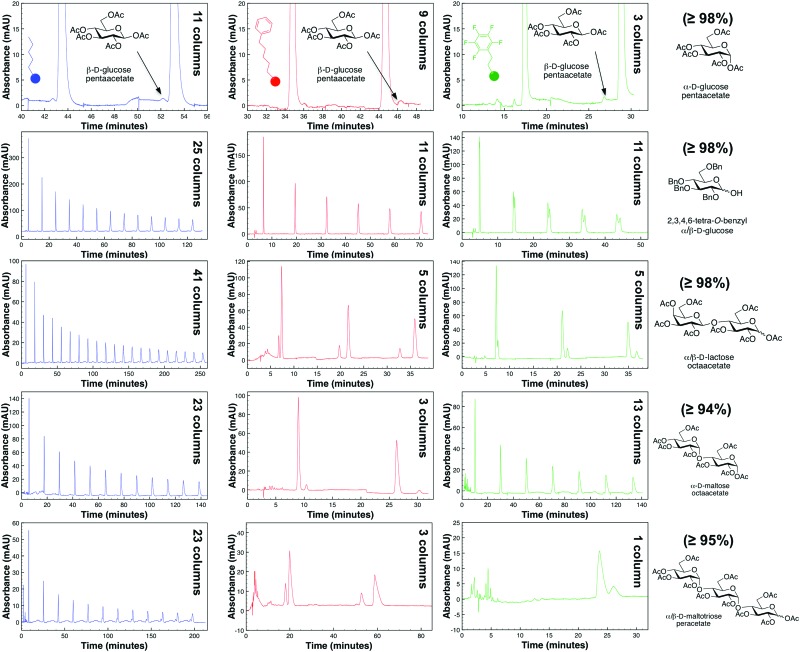
Comparison of various column chemistries in the purification of protected carbohydrates with our R-HPLC system with optimized conditions. Purity of each protected carbohydrate, as obtained from the manufacturer, is provided in parentheses.

From Fig. 2, interestingly, it was seen that both the PFP and phenyl hexyl stationary phases were superior in analyte separation as compared to the traditionally used alkyl-linked (C5) support. Specifically, a PFP stationary phase was observed to be best suited towards acyl-protected monosaccharides and benzyl (aromatic group) protected compounds (even with mutarotation^15^ in 60 °C), while a phenyl hexyl support was best suited for acyl-protected oligosaccharides. Additional chromatograms that illustrate other analytes and tables that discuss column efficiency are presented in the ESI.†

Based on these promising results with protected carbohydrate standards, this methodology was next used to purify a crude reaction mixture from our solution-phase automation platform.^16^ The toughest manual separations in our labs have been the products of chiral sugars linked to achiral components. Therefore, a reaction mixture resulting from glycosylation of a chiral thioglycoside^17^ with an achiral fluorous tag (see ESI† for more details) was subjected to our R-HPLC (Fig. 3) conditions with a PFP column given that this stationary phase was best suited for aromatic protecting group containing carbohydrates. After seven total/effective columns, the desired final product was successfully purified from undesired impurities, collected, dried, and fully characterized (ESI†).

**Fig. 3:**
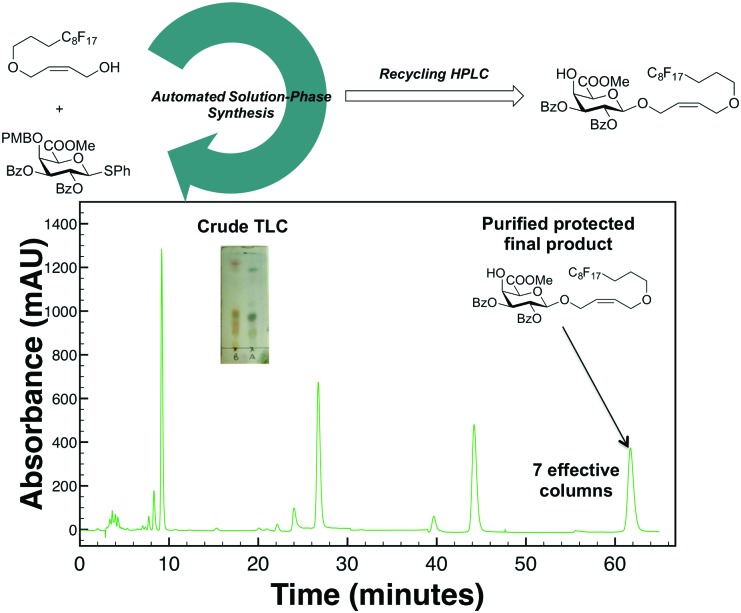
Purification of synthesized protected carbohydrate *via* automation solution-phase platform after fluorous solid-phase extraction with alternate-pump R-HPLC.

Herein the first analytical method to purify protected carbohydrates at levels of ≥99.5% is presented with a recycling high-performance liquid chromatography system. It was observed that the commonly used C5 stationary phase is far inferior to either a pentafluorophenyl stationary phase for the purification and fraction collection of protected monosaccharides or a phenyl hexyl stationary phase for protected oligosaccharides. This method and instrumentation can easily be adopted in any laboratory that already has an existing HPLC system (see ESI†) with only the addition of a 10-port switching valve and two identical columns. Purification at the protected stage is especially important given the relative ease of NMR identification of saccharides in this state; if the protected compound can be purified at levels of ≥99.5%, then deprotection can easily be performed without concerns of regio- or stereoisomer contamination in the final product. Whereas the current setup can easily purify the 1–5 mg commonly made through automated oligosaccharide synthesis^16,18^—enough for a variety of cell-based assays and microarrays—in 24 h or less when several effective columns (<10 for all compounds in this study) are needed, the method should be easily translated to a semi-preparative or preparative alternate-pump R-HPLC system to allow for larger scale purifications and continuous throughput with isocratic mode separations and thereby facilitate the acceptance by the biology and analytical chemistry communities of oligosaccharides from automated synthesis platforms.

We would like to acknowledge the National Institutes of Health (5U01GM116248-02 to NLBP and GM R01 24349-28 to MVN) and the Joan and Marvin Carmack Chair funds for partial support of this work.
