# Supplementary material for: Protocol for the purification of protected carbohydrates: toward coupling automated synthesis to alternate-pump recycling high-performance liquid chromatography
Source: Chem Commun (Camb). 2016 Oct 24;52(90):13253–6. doi: 10.1039/c6cc07584c (PMC5123635; doi:10.1039/c6cc07584c)
Supplement: Supplementary file 1 [file CC-052-C6CC07584C-s001.pdf]

Electronic Supplementary Material (ESI) for ChemComm.
This journal is © The Royal Society of Chemistry 2016

Electronic Supplementary Material for Chemical Communications

Protocol for the purification of protected carbohydrates: Toward coupling automated synthesis to alternate-pump recycling high-performance liquid chromatography

Gabe Nagy [a†], Tianyuan Peng [a†], Daniel E. K. Kabotso [a], Milos V. Novotny [a], and Nicola L. B. Pohl [a††]

[†] Both authors contributed equally

[††] Corresponding author

[a] Department of Chemistry, Indiana University, Bloomington, IN 47405

Email: npohl@indiana.edu

Phone: 812-855-0298

Fax: 815-855-8300

Supporting Information Table of Contents

Materials, reagents, conditions S2–S3

Alternate-pump R-HPLC schematic and conversion to alternate-pump R-HPLC from normal HPLC setup S4–S5

Acetonitrile versus methanol as organic modifier S6

Chromatograms S7–S10

Theoretical plates discussion S11–S12

Reaction conditions for automated glycosylation S13

Automated solution-phase synthesis procedure S14–S15

Characterization of purified compound from Figure 3 S16–S17

Materials, reagents, conditions

Column information
Luna® Perfluoro Phenyl (PFP) LC Columns (100Å, 4.6 mm × 250mm, 5-μm particle size), Luna® Phenyl Hexyl LC Columns (100Å, 4.6 mm × 250mm, 5-μm particle size) and Luna® C5 LC Columns (100Å, 4.6 mm × 250mm, 5-μm particle size) were acquired from Phenomenex, Inc. (Torrance, CA).

RED = phenyl hexyl stationary phase

BLUE = C5 stationary phase

GREEN = pentafluorophenyl stationary phase

Chemicals
HPLC-grade solvents were used for all experiments: water (H_2O), acetonitrile (ACN), and methanol (MeOH). Sigma Aldrich Co. (St. Louis, MO, USA) provided D-lactose octaacetate (≥ 98%) and 2,3,4,6 tetra-O-benzyl D-glucose (≥ 98%). Carbosynth (Berkshire, UK) was the source for 2-acetamido-1,3,4,6-tetra-O-acetyl-2-deoxy-β-D-glucopyranose (≥ 98%), 2-acetamido-1,3,4,6-tetra-O-acetyl-2-deoxy-α-D-glucopyranose (≥ 98%), 1,2,3,4,6-penta-O-acetyl-β-D-mannopyranose (≥ 98%), 1,2,3,4,6-penta-O-acetyl-α-D-mannopyranose (≥ 98%), α-D-maltose octaacetate (≥ 94%), and D-maltotriose peracetate (≥ 95%). 1,2,3,4,6-penta-O-acetyl-α-D-galactopyranose (≥ 98%), 1,2,3,4,6-penta-O-acetyl-β-D-galactopyranose (≥ 98%), 1,2,3,4,6-penta-O-acetyl-α-D-glucopyranose (≥ 98%), 1,2,3,4,6-penta-O-acetyl-β-D-glucopyranose (≥ 98%), β-D-maltose octaacetate (≥ 98%), and α-D-cellobiose octaacetate (≥ 98%) were purchased from Santa Cruz Biotechnology Inc. (Dallas, Texas, USA).

Instrument parameters
UV detection was at 225 nm for all acyl-protected compounds and at 254 nm for aromatic (benzyl/benzoyl) compounds. Flow rates of 1 mL/min at 60 °C were used for all compounds. No pH buffer control, because of potential hydrolysis, or mobile-phase additive salts, because of sample contamination that would require further desalting steps, were used in any mobile phase conditions. For method conversion from one that uses acetonitrile as an organic modifier to one that uses methanol, instead, it is important to mention that a 10% increase in organic modifier is necessary (ex. 50% acetonitrile to 60% methanol) because methanol has ~10% less eluotropic strength as compared to acetonitrile. This can be useful when the sample diluent cannot be altered based on solubility or limited sample quantity.

Experimental conditions

**Acylated monosaccharides**	**C5**	**Phenyl hexyl**	**PFP**
Mobile Phase	55/45 CH_3CN/H_2O	65/35 $MeOH/H_2O$	65/35 $MeOH/H_2O$
Sample diluent	50/50 CH_3CN/H_2O	60/40 $MeOH/H_2O$	60/40 $MeOH/H_2O$
Injection amount	200 µg	100 µg	100 µg

**Acylated disaccharides**	**C5**	**Phenyl hexyl**	**PFP**
Mobile Phase	55/45 CH_3CN/H_2O	65/35 $MeOH/H_2O$	65/35 $MeOH/H_2O$
Sample diluent	50/50 CH_3CN/H_2O	60/40 $MeOH/H_2O$	60/40 $MeOH/H_2O$
Injection amount	150 µg	150 µg	150 µg

**Acylated trisaccharides**	**C5**	**Phenyl hexyl**	**PFP**
Mobile Phase	55/45 CH_3CN/H_2O	65/35 $MeOH/H_2O$	65/35 $MeOH/H_2O$
Sample diluent	50/50 CH_3CN/H_2O	60/40 $MeOH/H_2O$	60/40 $MeOH/H_2O$
Injection amount	100 µg	100 µg	100 µg

**Benzyl monosaccharides**	**C5**	**Phenyl hexyl**	**PFP**
Mobile Phase	80/20 CH_3CN/H_2O	90/10 $MeOH/H_2O$	90/10 $MeOH/H_2O$
Sample diluent	75/25 CH_3CN/H_2O	75/25 CH_3CN/H_2O	75/25 CH_3CN/H_2O
Injection amount	100 µg	100 µg	100 µg

**Final compound (Figure 3)**	**PFP**
Mobile Phase	87.5/12.5 $MeOH/H_2O$
Sample diluent	85/15 $MeOH/H_2O$

Table S1. Experimental conditions used with alternate-pump R-HPLC.

Alternate-pump R-HPLC schematic and conversion to alternate-pump R-HPLC from normal HPLC setup

Alternate-pump R-HPLC procedure and switching times

The alternate-pump R-HPLC setup used in this study consisted of a Dionex P680 HPLC instrument with a PDA-100 UV-detector, an autosampler, and 10-port switching valve (Vici Valco Instruments, Houston, TX). At the core, this system utilized two identical columns that are connected with a 10-port switching valve with two positions (A and B), along with a UV-detector. This system begins in position A and after a compound is injected, it will flow through the first column until it hits the UV-detector at its retention time (t_r). After the compound is halfway through the second column (1.5 t_r), the switching valve will change from position A to B. Once the compound travels another column length and is halfway through the first column (2.5 t_r), the valve will switch from position B to A, where once it is out of the first column, it will once again hit the UV-detector. This process is repeated for as many effective columns as necessary to provide the desired separation and permit fraction collection since the UV-detector employed is non-destructive in nature. *To reiterate, two identical columns and column chemistries must be used in this alternate-pump R-HPLC system such that the retention times and switching times are uniform.*

Changing from a normal HPLC to alternate-pump R-HPLC setup

The only parts required to adapt an existing HPLC to a alternate-pump R-HPLC system are: one 10-port switching valve, a second identical column, and a non-destructive detector such as UV-Vis or refractive index (if not already installed). As mentioned above, a 10-port switching valve (the one used in this study has pressure limits of <5000 psi) operates in two A/B positions and the diagram below indicates where the PEEK tubing connections are placed (must connect the system starting from position A). Additional PEEK tubing and corresponding ferrules will also be needed to connect all 10-ports and two columns together.

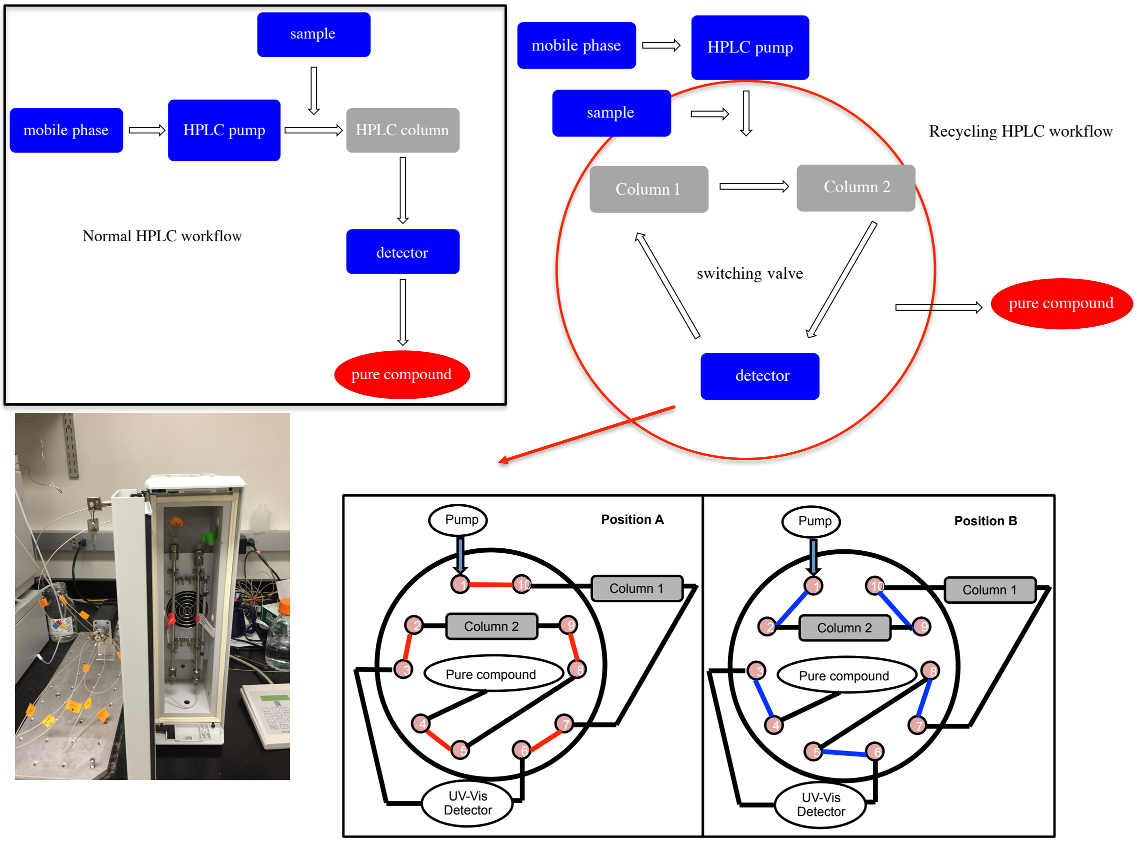

Figure S1. Conversion of normal HPLC to alternate-pump R-HPLC system, along with schematic of 10-port switching valve between positions A and B.

Acetonitrile versus methanol as organic modifier

As mentioned in the manuscript, it is observed that methanol serves as a superior organic modifier as compared to acetonitrile for π-bonded stationary phases when the analytes also contain π-bonds. This is explained by the suppression of π- π interactions when acetonitrile is used because of its carbon-nitrogen triple bond so that the analyte is unable to interact as well with the stationary phase.

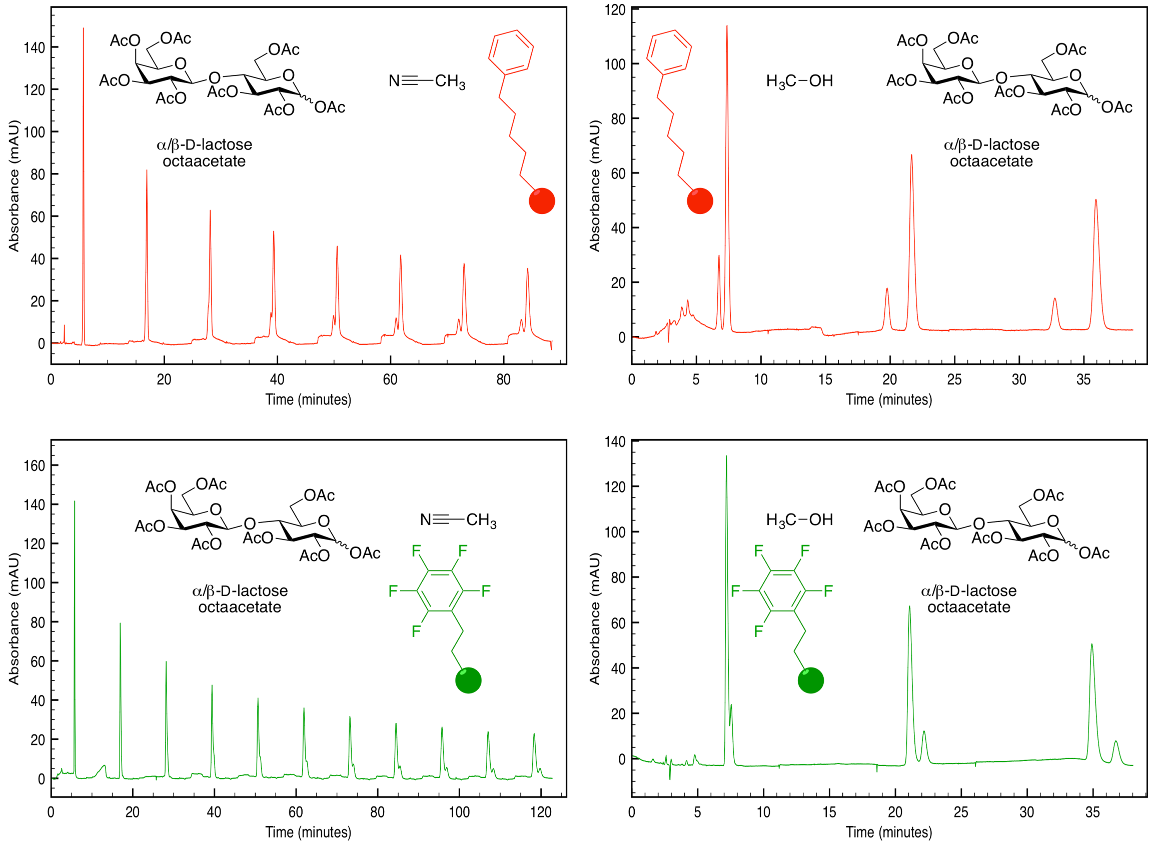

Figure S2. Mobile phase screening for phenyl hexyl and pentafluorophenyl reversed-phase columns with an alternate-pump R-HPLC setup of α/β-D-lactose octaacetate (≥ 98% purity). It is seen that after 5 total columns with methanol as the organic modifier with either the PFP or phenyl hexyl stationary phase, the α/β anomers are separated, which is much faster than when acetonitrile is used as the organic modifier.

Chromatograms

For all chromatograms shown, the percentage listed above the structures indicates the proportion that was added of each analyte. The purity (as given from the manufacturer) is listed on page S2 as well as in the figure caption.

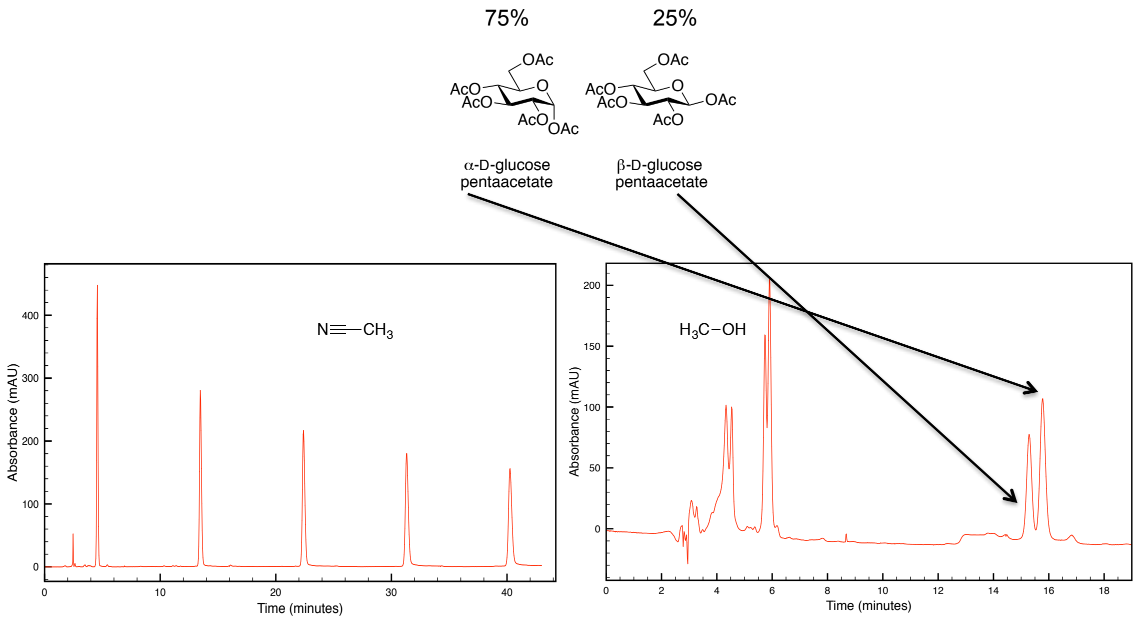

Figure S3. Mixture of 75% α-D-glucose pentaacetate (≥ 98% purity) and β-D-glucose pentaacetate (≥ 98% purity) with changing the organic modifier from acetonitrile to methanol. It can be seen that no separation is achieved with acetonitrile, but two peaks are clearly present when changing to methanol.

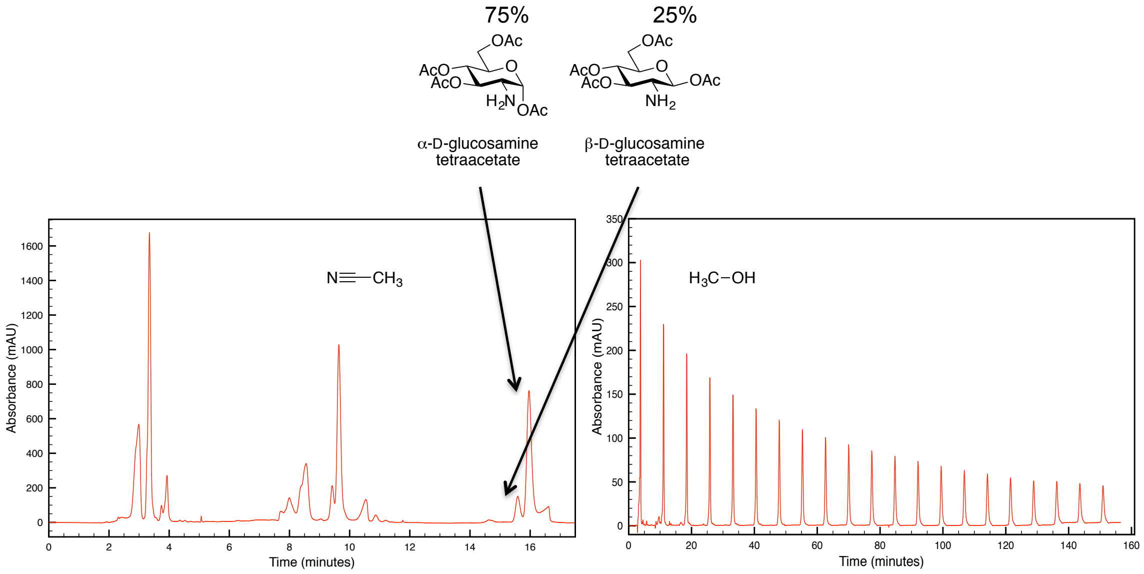

Figure S4. Mixture of 75% α-D-glucosamine tetraacetate (≥ 98% purity) and β-D-glucosamine tetraacetate (≥ 98% purity) with changing the organic modifier from acetonitrile to methanol. The opposite phenomenon is observed as with all other mobile phase conditions. Instead, when methanol is used, no separation is achieved. This is hypothesized to be from methanol causing constant protonation/deprotonation of the amine group that limits separation, as opposed to no such occurrence when acetonitrile is used. Two peaks are evident on the left after a single column (although coeluting), while no separation is observed on the right.

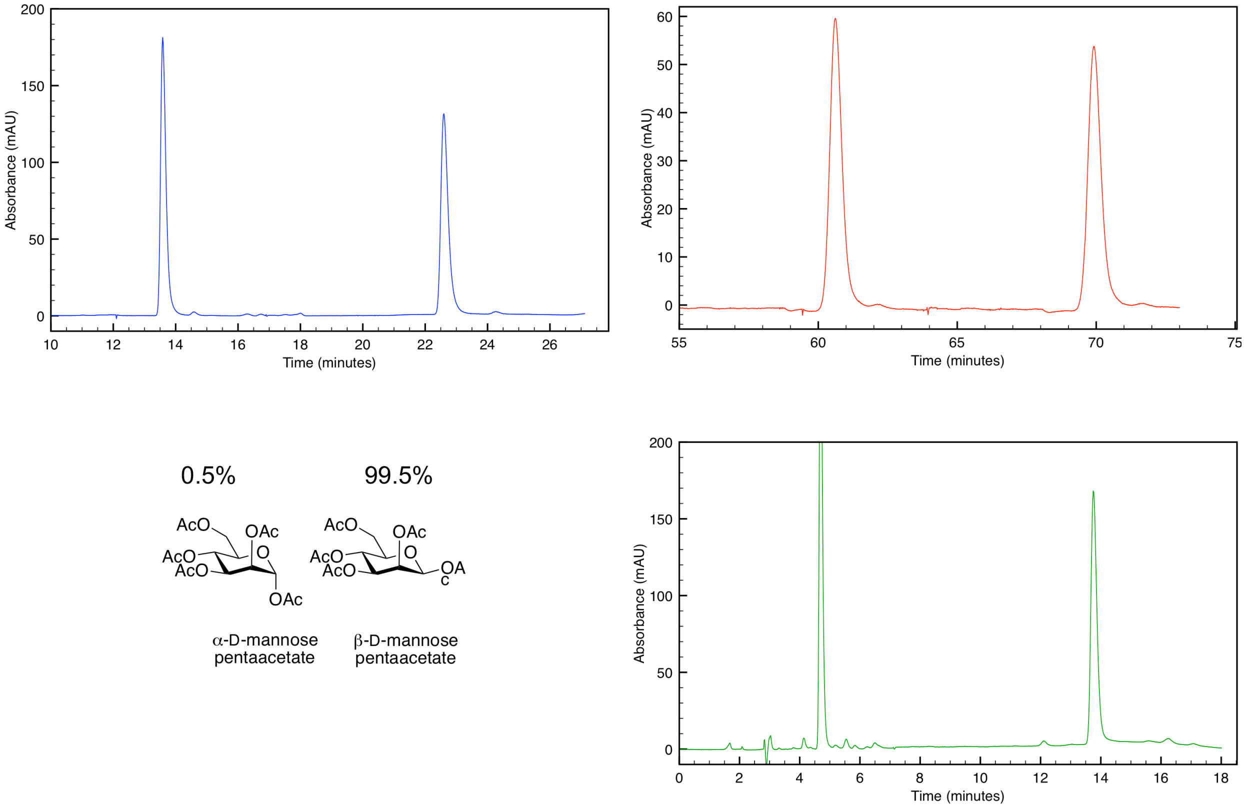

Figure S5. Cross column comparison for a mixture of 0.5% α-D-mannose pentaacetate (≥98% purity) and 99.5% β-D-mannose pentaacetate (≥ 98% purity). It is seen that the pentafluorophenyl stationary phase with a methanol-containing mobile phase is optimal for separation of protected monosaccharides for low-level impurities.

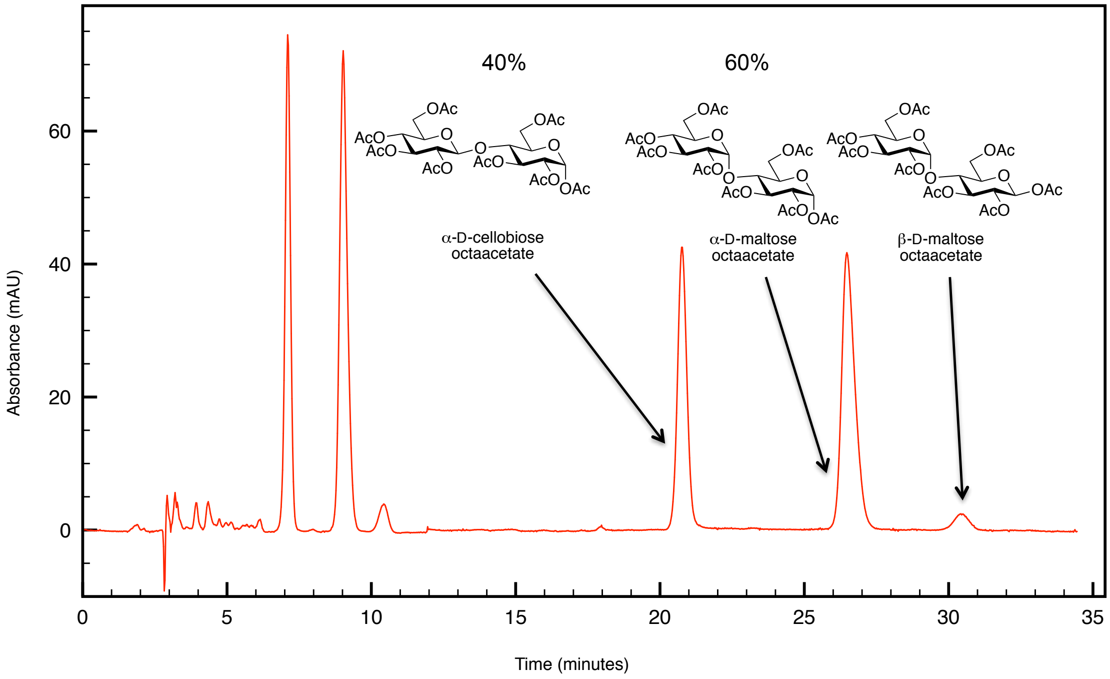

Figure S6. Stereoisomer separation on a phenyl hexyl stationary phase. It is seen that a mixture of 40% α-D-cellobiose octaactate (≥98% purity) and 60% α-D-maltose octaacetate (≥ 94% purity) can be easily separated in one column with this stationary phase and mobile phase combination after 3 total columns. The peaks between 5 and 10 minutes represent the separation after 1 total column.

Theoretical plates discussion

$$Theoretical\ plates\ (N) = 5.54 \times \frac{t_r{}^2}{0.5w^2}$$

$$\frac{Theoretical\ plates}{Meters\ of\ effective\ column}\left(\frac{N}{m}\right) = 5.54 \times \frac{t_r{}^2}{0.5w^2} \times \frac{1}{m}$$

	C5	Phenyl hexyl	Pentafluorophenyl
α/β-D-galactose pentaacetate	5 columns<br>24.16 min	9 columns<br>45.53 min	1 column<br>5.39 min
α/β-D-glucose pentaacetate	11 columns<br>53.07 min	9 columns<br>44.66 min	3 columns<br>16.98 min
α/β-D-mannose pentaacetate	3 columns<br>13.58 min	9 columns<br>43.23 min	1 column<br>5.51 min
α/β-D-glucosamine tetraacetate	5 columns<br>17.18 min	n/a*	3 columns<br>11.23 min
α/β-D-maltose octaacetate	n/a	1 column<br>8.98 min	13 columns<br>133.19 min
α/β-D-lactose octaacetate	n/a	3 columns<br>21.68 min	5 columns<br>34.91 min

Table S2. Column efficiency for stationary phases used in recycling-high performance liquid chromatography where both number of effective columns and retention time to reach baseline separation of protected carbohydrates is provided. * indicates no separation with methanol as organic modifier, but effective separation with acetonitrile. Acetylated monosaccharide and disaccharide conditions are the same as those listed in Table S1.

C5

**Acetylated anomer pairs**	**Peak width (min)**	**T_r (min)**	**Effective columns**	**N/m**
Galactose	0.25	24.16	5	41392
Glucose	0.57	53.07	11	17463
Mannose	0.18	13.58	3	42044
Glucosamine	0.21	17.18	5	29662
Maltose	n/a	n/a	n/a	n/a
Lactose	n/a	n/a	n/a	n/a

Phenyl hexyl

**Acetylated anomer pairs**	**Peak width (min)**	**T_r (min)**	**Effective columns**	**N/m**
Galactose	0.42	45.53	9	28935
Glucose	0.4	44.66	9	30693
Mannose	0.39	43.23	9	30253
Glucosamine	n/a	n/a	n/a	n/a
Maltose	0.27	8.98	1	24513
Lactose	0.39	21.69	3	22847

PFP

**Acetylated anomer pairs**	**Peak width (min)**	**T_r (min)**	**Effective columns**	**N/m**
Galactose	0.13	5.39	1	38094
Glucose	0.27	16.98	3	29214
Mannose	0.14	5.51	1	34326
Glucosamine	0.2	11.23	3	23289
Maltose	1.52	133.19	13	13088
Lactose	0.47	34.91	5	24451

Table S3. Raw data and calculations for theoretical plates per meter from this alternate-pump R-HPLC system. N/m is defined as theoretical plates per meters of effective column (equation given on page S14), while n/a denotes that separation of anomers was not possible with the conditions used.

Reaction conditions for automated glycosylation

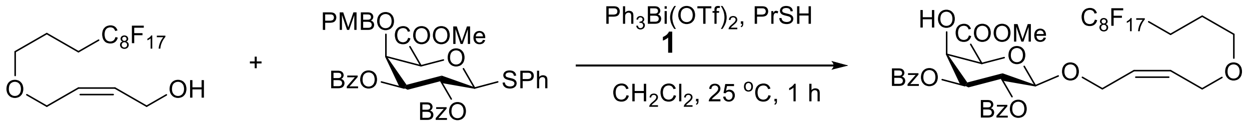

Table: Automated solution phase synthesis of compound 4

Step	Task	Reagent/Operation	Operation Time
1	Glycosylation	1.5 equiv donor (206 mg, 328 μmol) in 2.0 mL CH_2Cl_2, 1.0 equiv Ftagged acceptor (90.0 mg, 164 μmol) in 1.0 mL CH_2Cl_2, 1.5 PrSH (12.5 mg, 328 μmol) in 0.6 mL CH_2Cl_2,1.5 equiv $Ph_2Bi(OTf)_2$ (242 mg, 328 μmol) in 3.0 mL CH_2Cl_2, 25 °C	60 min
2	TLC	0.1 mL of crude reaction mixture withdrawn	
3	Quenching	0.1 mL of Et_3N, 25 °C	
4	Transfer	Crude reaction mixture withdrawn into product vial Reaction vial rinsed (3 mLx2 CH_2Cl_2) and transfer into product vial	
5	Evaporation	Bench top: 43 °C	
6	FSPE	Bench top: Loading 90% DMF/H_2O fluorophobic elution: 80% MeOH/H_2O Fluorophilic elution: 100% CH_3COCH_3	

Figure S7. Automated glycosylation conditions with quenching, followed by removal of the crude product from the automation platform for solvent removal and fluorous solid-phase extraction prior to R-HPLC. Bz = benzoyl, Ph = phenyl, Me = methyl, Pr = propyl.

Automated solution-phase synthesis procedure

To synthesize compound **4** via glycosylation using the automated solution-phase synthesis platform, the samples were prepared as follows.

Compounds **1** (242 mg, 328 µmol, 1.5 equiv; in 3.0 mL anhydrous CH_2Cl_2), **2** (90.0 mg, 164 µmol, 1.0 equiv; in 1.0 mL anhydrous CH_2Cl_2), **3** (206 mg, 328 µmol, 1.5 equiv; in 2.0 mL anhydr. CH_2Cl_2), and propanethiol (12.5 mg, 328 µmol, 1.5 equiv in 0.6 mL anhydrous CH_2Cl_2) in separate 8 mL-vials were placed on the reagent block as shown in the diagram below.

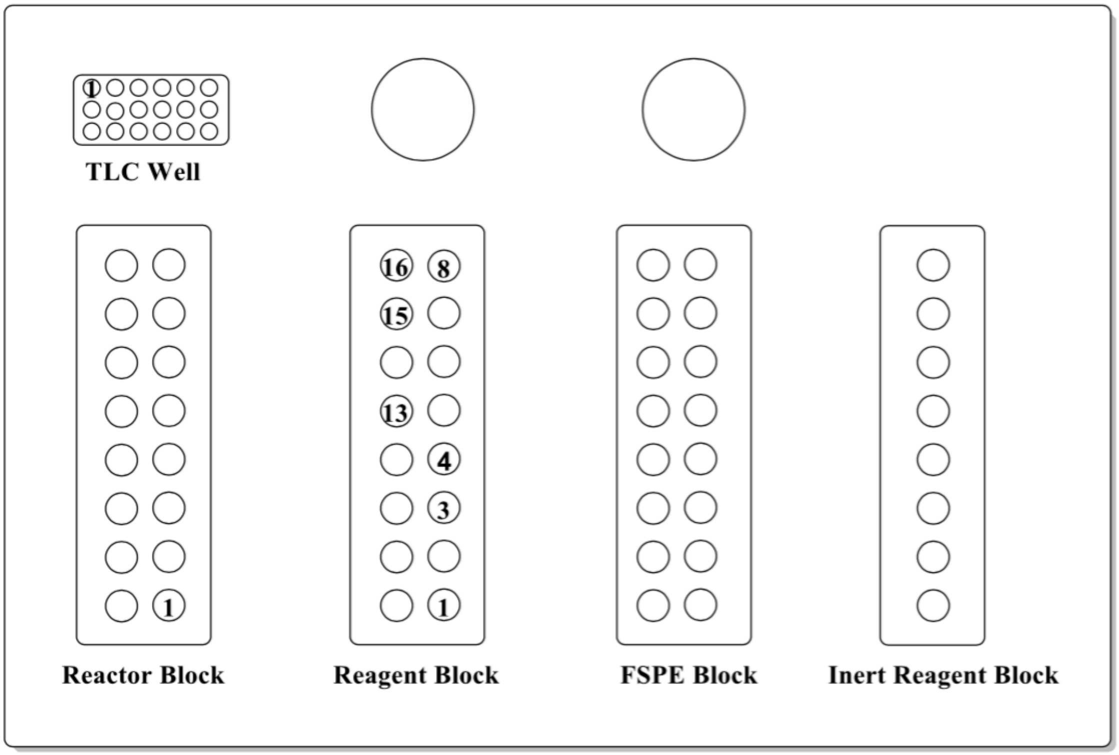

Reactor Block Positions

1. Reaction Vial One

Reagent Block Positions

1. Alkene Tag (**2**, Acceptor)
3. Propanethiol (PrSH)
4. Bismuth Promoter (**1**)
8. Final position for product
13. Triethylamine (TEA)
15. Donor (**3**)
16. Dichloromethane (DCM)

Figure S8. Schematic of automated solution-phase platform as used in this experiment. Two needles (not drawn) can deliver solutions (left-side needle up to 5 mL, right-side needle up to 10 mL) between the diagrammed locations.

This particular automated solution-phase synthesis run was originally used to test the ability of a bismuth promoter (reference 17 in manuscript) to mediate the glycosylation of a phenyl thioglycoside with a fluorous tag rather than another sugar building block. It was chosen as good test example for purification as it involves the addition of a chiral building block to an achiral component (the fluorous tag).

After the preparation of the samples, the reactant vials were cleaned, dried and purged with argon by following the standard cleaning cycle protocol as previously reported (reference 16 in manuscript). The coupling of the donor **3** and acceptor **2** mediated by bismuth promoter **1** in the presence of propanethiol additive was carried out as follows.

The donor (**3**) solution was transferred quantitatively from reagent block position 15 using the left needle into reactor vial position 1 on the reactor block. Without rinsing the needle, it was used to transfer the acceptor (**2**) solution from the reagent vial position 1 into the donor solution located in reaction vial 1. This move was followed by the transfer of the propanethiol from the reagent vial position 3 into the donor and acceptor in the reaction vial 1. The needle was then rinsed with toluene before it was used to transfer the promoter (**1**) solution to prevent premature interaction between the promoter and propanethiol. After the transfer, the needle was rinsed and the platform was vortexed at a speed of 500 rpm. The vortexing was continued for 1 h after which a 0.1 mL aliquot of the reaction was transferred into TLC well 1. TLC analysis confirmed the completion of the reaction. The reaction was quenched by the transfer of neat Et_3N (0.1 mL) from the reagent block position 13 into reaction vial 1. The reaction mixture was then transferred quantitatively from reaction vial 1 into a clean 8-mL vial at reagent block position 8. Reaction vial 1 was rinsed by transferring CH_2Cl_2 from reagent block position 16 into reaction vial 1 and then transferred back into the product collection vial at position 8 of the reagent block. After removal from the automation platform, the collected product was concentrated under reduced pressure followed by coevaporation with toluene to azeotropically remove traces of Et_3N. To remove gross impurities prior to alternate-pump R-HPLC purification, the crude material was dissolved in 90% DMF in H_2O and loaded onto a fluorous silica gel column (5 g), which was previously washed with DMF (2 mL) and equilibrated with 80% MeOH in H_2O (12 mL). Fluorophobic elution to remove all non-fluorous compounds was done using 80% MeOH in H_2O. Fluorophilic elution using 100% acetone furnished the fluorous compound, which was concentrated under reduced pressure followed by azeotropic removal of water using toluene. Following fluorophilic elution, the crude material was subjected to a short silica pipet column (ethyl acetate/hexane 20/80) to ensure no other grossly impure compounds would be loaded onto the alternate-pump R-HPLC system. Following this, the material was then subjected to alternate-pump R-HPLC, where the major peak, as shown in Figure 3 of the manuscript, is desired product **4**.

Characterization of purified compound from Figure 3

HRMS calculated M+Na⁺ = 969.1544 *m/z*
HRMS experimental M+Na⁺ = 969.1544 *m/z*

^{1}H NMR (500 MHz, $CDCl_3$) δ 7.99, 7.98, 7.97, 7.97, 7.96, 7.96, 7.52, 7.51, 7.50, 7.50, 7.39, 7.39, 7.38, 7.38, 7.38, 7.37, 7.36, 7.26, 5.79, 5.77, 5.77, 5.76, 5.71, 5.70, 5.70, 5.69, 5.69, 5.68, 5.67, 5.67, 5.66, 5.66, 5.65, 5.65, 5.65, 5.64, 5.64, 5.64, 5.63, 5.63, 5.63, 5.62, 5.36, 5.36, 5.35, 5.34, 4.78, 4.77, 4.68, 4.68, 4.68, 4.67, 4.67, 4.67, 4.67, 4.46, 4.46, 4.45, 4.37, 4.37, 4.35, 4.35, 4.34, 4.34, 4.33, 4.32, 4.02, 4.01, 4.01, 4.00, 3.97, 3.96, 3.96, 3.84, 3.39, 3.38, 3.38, 3.37, 3.36, 3.36, 3.35, 3.35, 3.34, 3.34, 2.62, 2.61, 2.20, 2.18, 2.17, 2.16, 2.15, 2.13, 2.13, 2.13, 2.12, 2.12, 2.11, 2.11, 2.10, 2.10, 2.09, 2.06, 2.05, 2.04, 2.04, 2.03, 1.83, 1.82, 1.81, 1.81, 1.81, 1.80, 1.80, 1.80, 1.79.

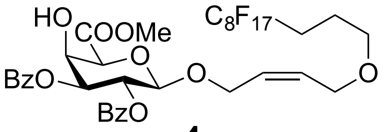

4

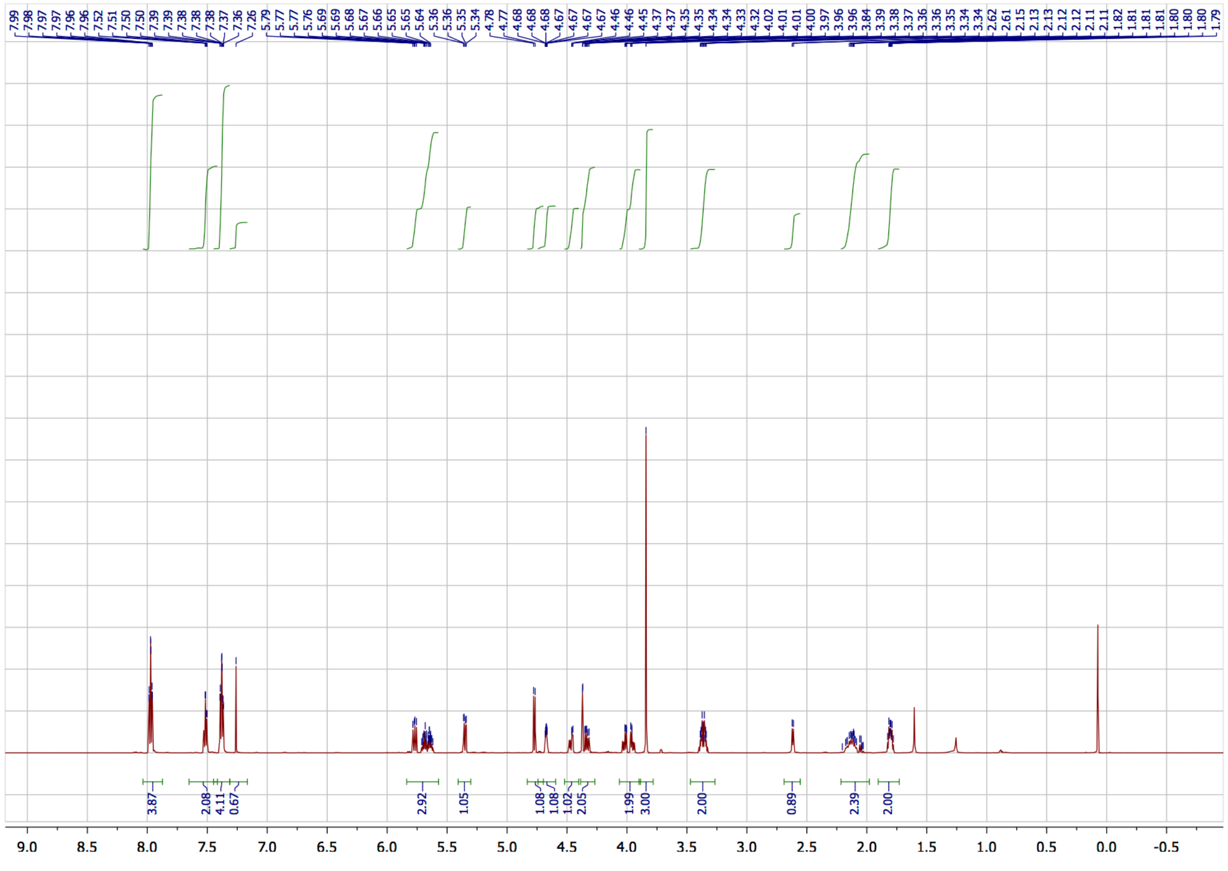

Figure S9. ^{1}H NMR in $CDCl_3$.

^{13}**C NMR (126 MHz, CDCl$_3$)** δ 166.63, 164.74, 164.14, 132.54, 132.24, 129.71, 128.88, 128.70, 128.36, 127.84, 127.47, 127.37, 126.46, 98.78, 76.24, 76.19, 75.99, 75.74, 72.91, 72.50, 68.11, 67.70, 67.37, 65.53, 63.64, 51.70, 27.14, 26.96, 26.78, 19.76, 0.00.

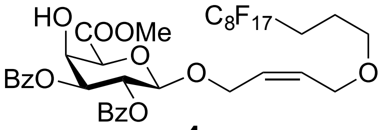

4

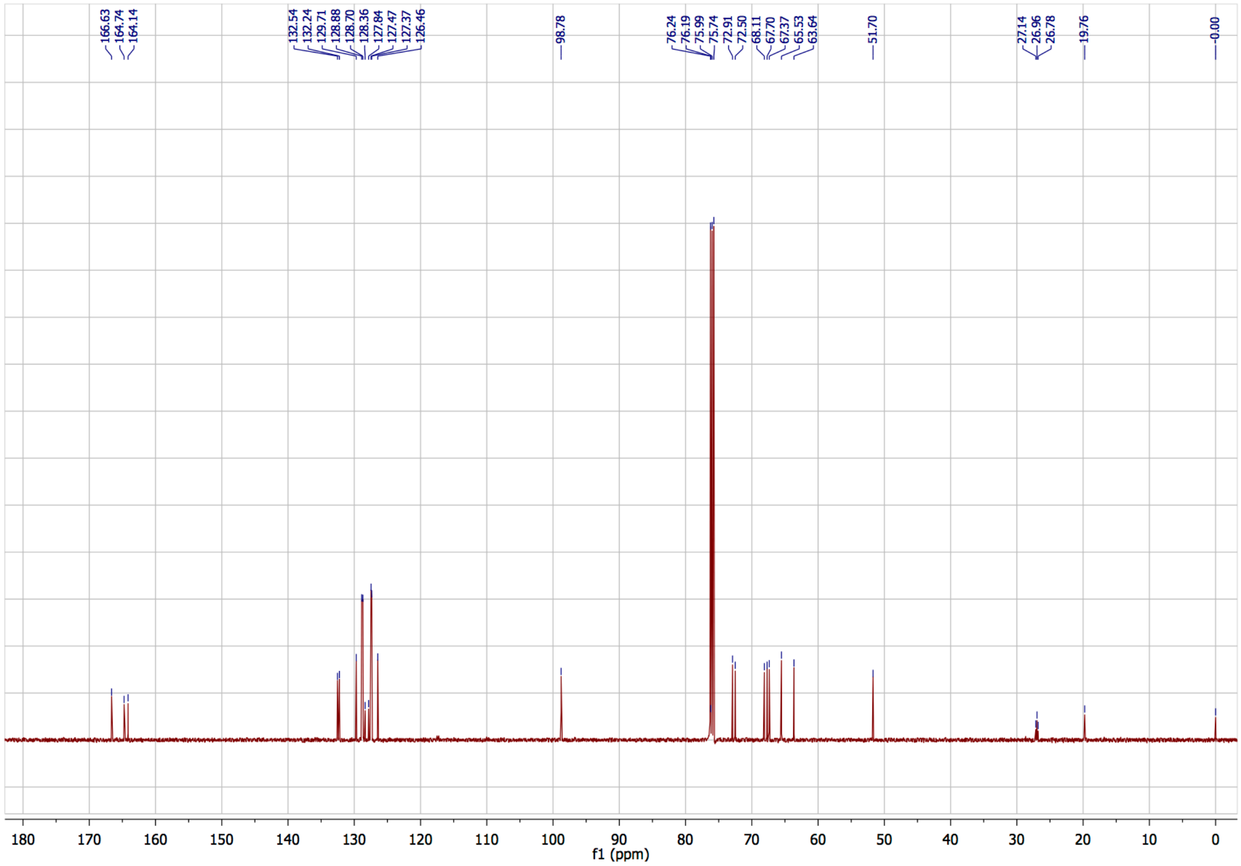

Figure S10. ^{13}C NMR in CDCl$_3$.
